# Obstructive Sleep Apnea Syndrome, Objectively Measured Physical Activity and Exercise Training Interventions: A Systematic Review and Meta-Analysis

**DOI:** 10.3389/fneur.2018.00073

**Published:** 2018-02-22

**Authors:** Monique Mendelson, Sébastien Bailly, Mathieu Marillier, Patrice Flore, Jean Christian Borel, Isabelle Vivodtzev, Stéphane Doutreleau, Samuel Verges, Renaud Tamisier, Jean-Louis Pépin

**Affiliations:** ^1^1HP2 Laboratory, University Grenoble Alpes, Grenoble, France; ^2^U1042, INSERM, Grenoble, France; ^3^AGIR à Dom. Association, Meylan, France; ^4^Grenoble Alps University Hospital, Grenoble, France

**Keywords:** obstructive sleep apnea, physical activity, exercise training, randomized controlled trials, systematic review, meta-analysis

## Abstract

A systematic review of English and French articles using Pubmed/Medline and Embase included studies assessing objective physical activity levels of obstructive sleep apnea (OSA) patients and exploring the effects of exercise training on OSA severity, body mass index (BMI), sleepiness, and cardiorespiratory fitness [peak oxygen consumption (VO2peak)]. Two independent reviewers analyzed the studies, extracted the data, and assessed the quality of evidence. For objective physical activity levels, eight studies were included. The mean number of steps per day across studies was 5,388 (95% CI: 3,831–6,945; *p* < 0.001), which was by far lower than the recommended threshold of 10,000 steps per day. For exercise training, six randomized trials were included. There was a significant decrease in apnea–hypopnea-index following exercise training (mean decrease of 8.9 events/h; 95% CI: −13.4 to −4.3; *p* < 0.01), which was accompanied by a reduction in subjective sleepiness, an increase in VO2peak and no change in BMI. OSA patients present low levels of physical activity and exercise training is associated with improved outcomes. Future interventions (including exercise training) focusing on increasing physical activity levels may have important clinical impacts on both OSA severity and the burden of associated co-morbidities. Objective measurement of physical activity in routine OSA management and well-designed clinical trials are recommended.

Registration # CRD42017057319 (Prospero).

## Introduction

Obstructive sleep apnea (OSA) is a common sleep disorder characterized by repeated episodes of apnea and hypopnea during sleep. Sleep fragmentation and chronic intermittent hypoxia induce intermediate mechanisms such as activation of the sympathetic nervous system (1), oxidative stress and systemic inflammation which contribute to cardiometabolic morbidity (2).

Physical activity is defined as “any bodily movement produced by the skeletal muscles that results in increased energy expenditure” (3) and is currently seen as one of the most powerful health-promoting behaviors (4). It has been associated with improved sleep and a bidirectional relationship between sleep and physical activity has been assumed (5). These observations are based on case–control studies showing that adults who report low-sleep quality presented lower levels of physical activity than similar adults without sleep complaints (6). For instance, adults with OSA are less likely to be active than adults without sleep apnea (7) and longitudinal and cross-sectional data suggest a reduced prevalence and incidence of OSA in those who exercise regularly (8). The low levels of physical activity observed in OSA patients have been attributed to the fatigue and somnolence they experience, the excess weight, and low energy that characterize the clinical presentation of OSA (9) and associated neurocognitive alterations [see for review: (10)].

Exercise training is a “subset of physical activity that is planned, structured, and repetitive” (3). In patients with OSA, exercise training as an adjunct to continuous positive airway pressure (CPAP) treatment has received growing interest in recent years. Regular physical activity has been associated with body weight maintenance (11), with reductions in blood pressure (12), and with the prevention of cardiovascular disease (13), therefore it may constitute a useful means to reduce cardiovascular and metabolic risk factors and co-morbidities associated with OSA.

The main objective of this systematic review and meta-analysis is to quantify objectively measured physical activity levels in OSA patients and to explore the effects of exercise training on OSA disease indices [apnea–hypopnea index (AHI) and Epworth sleepiness scale (ESS)] as well as on body mass index (BMI) and cardiorespiratory fitness [peak oxygen consumption (VO2peak)]. A secondary aim is to explore the effects of CPAP or exercise interventions on objectively measured physical activity levels.

## Materials and Methods

The Preferred Reporting Items for Systematic Reviews and Meta-analyses statement and recommendations were followed for this meta-analysis (http://www.prisma-statement.org) (14) (see Table S1 in Supplementary Material for PRISMA checklist) and the trial was registered on the Prospero registry (# CRD42017057319).

### Search Strategy and Data Sources

A systematic literature review was conducted to identify manuscripts which investigated physical activity levels and effects of exercise training in OSA patients. The web-based literature search included PubMed/MEDLINE and Embase databases. Search terms were selected to reflect the condition and outcome parameters. Search terms included a combination of text word terms and medical subject headings (MeSH) or Emtree terms (see Supplementary Material for sample search strategy). For the condition, search terms included: “sleep apnea syndromes” (MeSH) OR “sleep apnea, obstructive” (MeSH) OR “sleep disordered breathing.” For objectively measured physical activity, search terms included: “actigraphy” (MeSH), “exercise” (MeSH), “sports” (MeSH), “walking” (MeSH), “accelerometer,” “actigraph,” “physical activity.” For exercise training, search terms included: “exercise” (MeSH), exercise therapy (MeSH), “rehabilitation” (MeSH), exercise training, aerobic exercise, physical activity. Terms were searched in all possible combinations using Boolean Logical operators (AND, OR, NOT). Additionally, a manual search of bibliographies of included articles was conducted to identify relevant references which may not have been found by the automated search. Obtained references were indexed and managed using EndNote X7.

### Eligibility Criteria

The following criteria were required for selection: (1) original research investigations; (2) conducted in humans; (3) conducted in adults; (4) include patients diagnosed with OSA of at least mild severity (AHI ≥ 5 events/h) based on polysomnography (PSG) or polygraphy. Articles in English and French only were retained. For physical activity, studies were included if they reported objectively measured physical activity levels, quantified as steps per day. The number of steps per day of OSA patients was compared with a threshold value of 10,000 steps per day (15). For exercise training, studies were included if they were a randomized controlled trial (RCT) specifically addressing the effects of an exercise training program in patients with sleep apnea (at least 75% of the sample were OSA), with an intervention program of at least 3 weeks duration, reporting pre- and post-intervention mean and SD or standard error of AHI or mean change and SD, published in a peer-review journal up to February 2017. Exclusion criteria included any studies not meeting all criteria described above.

### Data Items

#### Reviewing Procedure and Data Extraction

Database searches were first conducted in February 2017. All obtained references were reviewed, and if retained, data extraction was conducted. The first level of review was title and abstract screening. Irrelevant references were removed. Potentially relevant studies were further assessed by obtaining and reading the full text and checking again the pre-specified eligibility criteria.

Titles and/or abstracts of studies retrieved using the search strategy and those from additional sources were screened independently by two review authors (Monique Mendelson and Mathieu Marillier) to identify studies that potentially met the inclusion criteria outlined above. The full text of these potentially eligible studies were retrieved independently and assessed for eligibility by two review team members.

**Figure 1:**
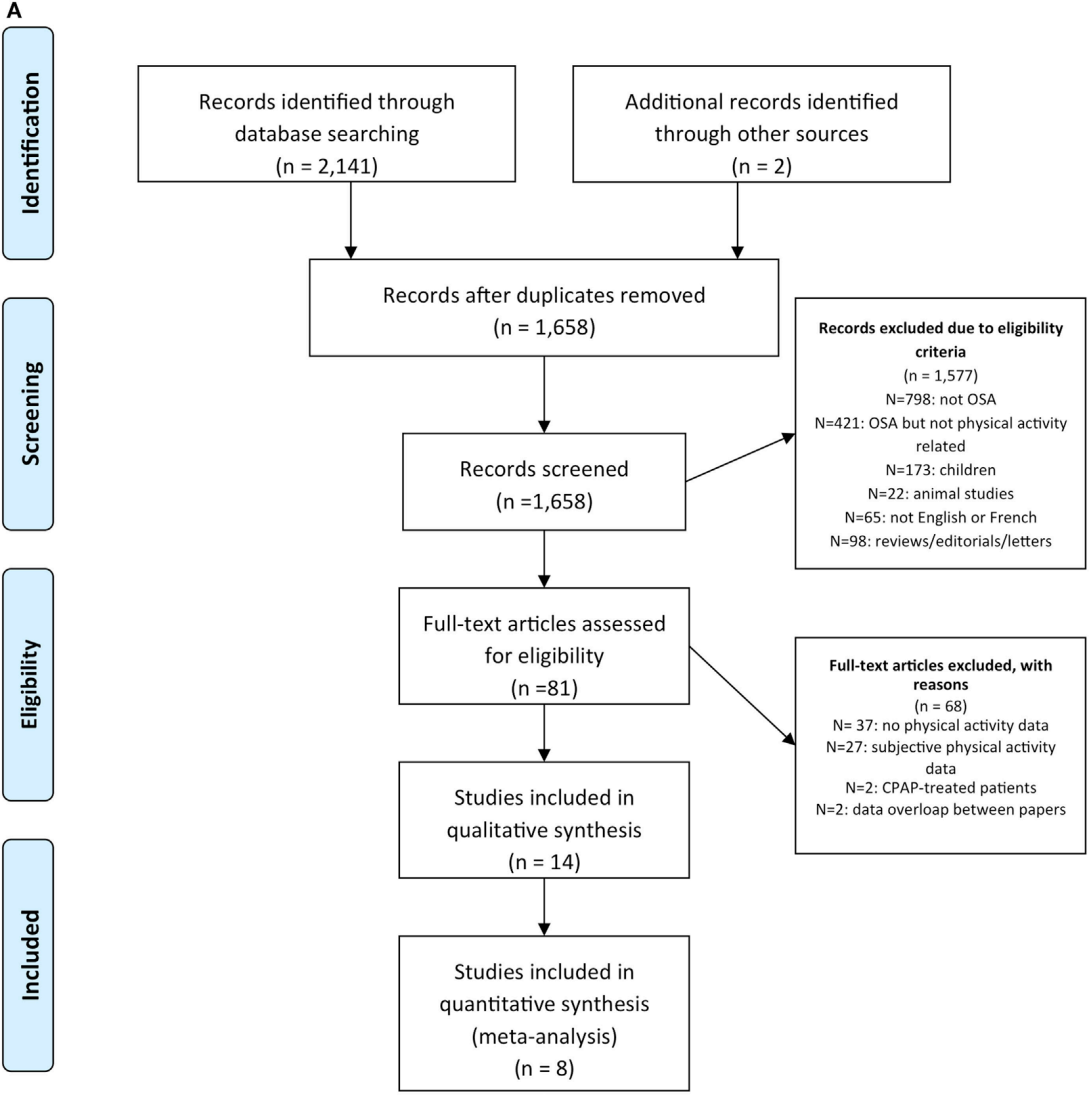

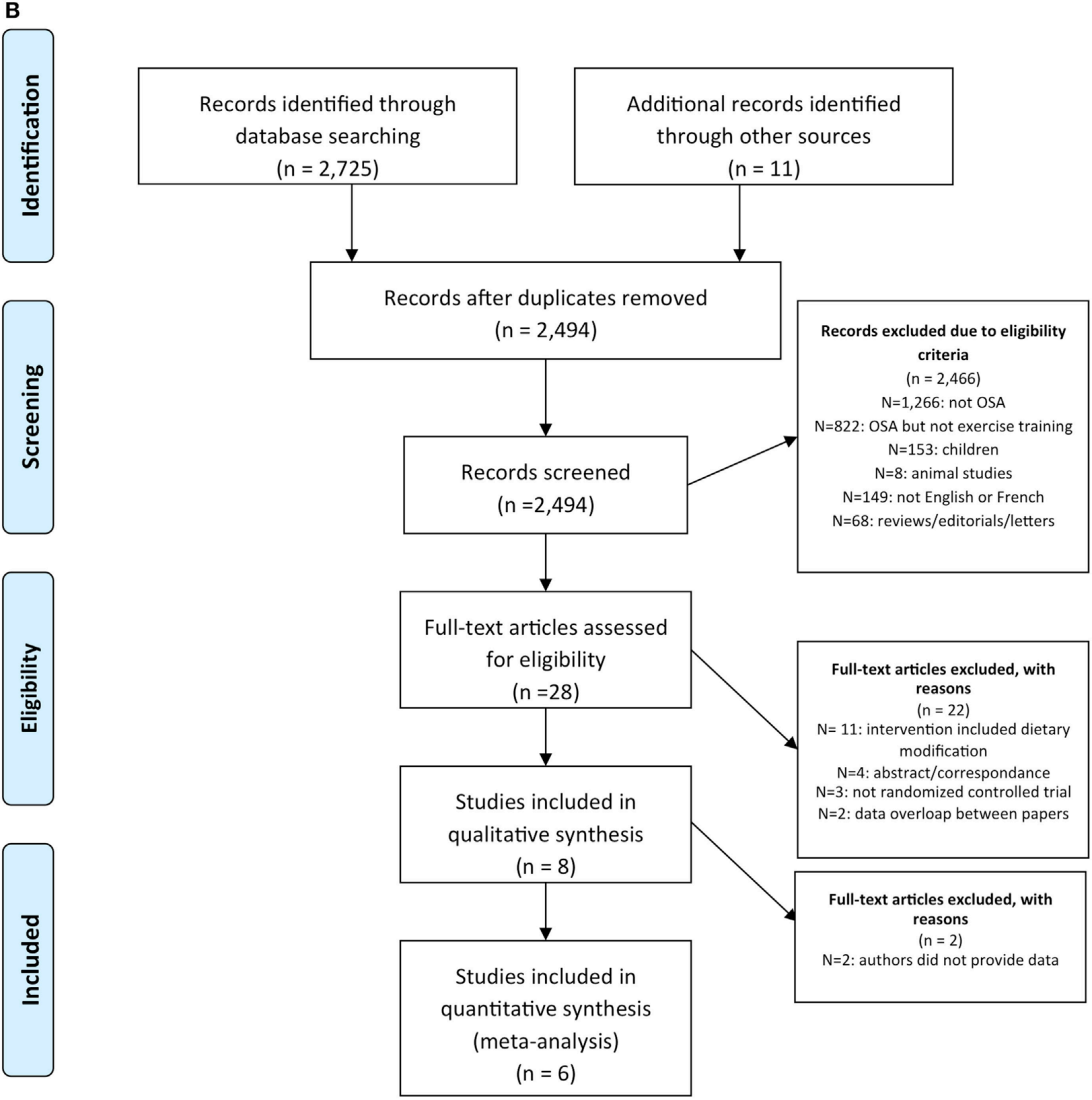
Prisma flow chart of articles identified and evaluated during the study selection process for **(A)** physical activity and **(B)** exercise training.

**Figure 2:**
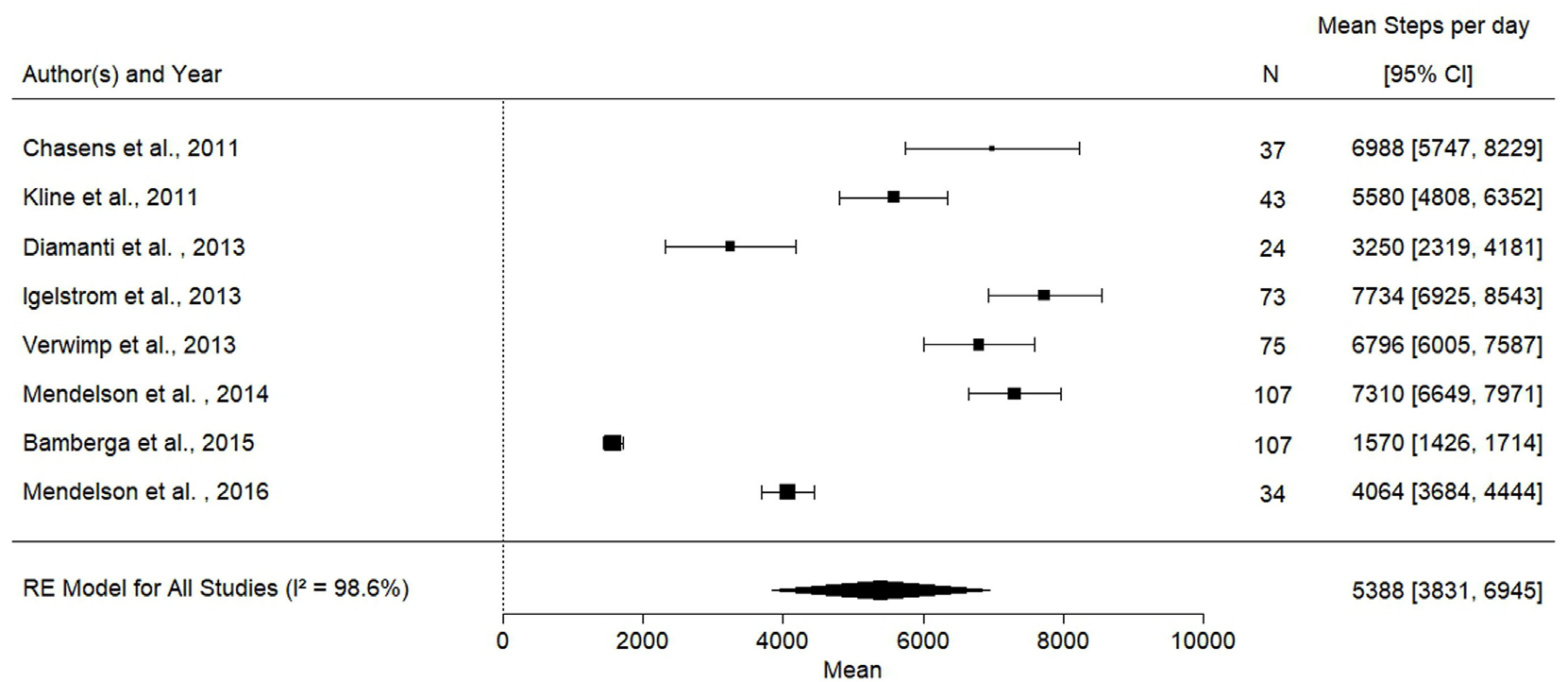
Forest plot for mean physical activity levels in obstructive sleep apnea patients. Ref (7, 16–22).

For each reference, the following variables were systematically extracted and entered into a summary table: (1) author, year; (2) participants; (3) AHI cutoff; (4) sample size; (5) age; (6) BMI; (7) study design; (8) outcomes; and (9) main findings. A summary of the studies screened, assessed for eligibility, and included is presented in Figures 1 and 2.

### Methodological Quality Assessment

The quality of the studies was peer-reviewed by Monique Mendelson and Mathieu Marillier using a modified version of the Newcastle-Ottawa Scale (NOS) for observational studies (23) and the Jadad scale for randomized trials (24). Disagreements were resolved by consensus. For the NOS, a system of points (stars) was given to the eligible categories: sample selection criteria, comparability on the basis of the design or analysis, and evaluation of outcome. The scale scores ranged from 0 to 6. Studies with scores above the median were classified as high-quality studies (25).

The Jadad score (24) was used to assess the methodological quality of controlled trials. Studies were scored according to the presence of three key methodological features of clinical trials, specifically randomization, masking, and accountability of all patients, including withdrawals. Blinding of participants to exercise interventions is virtually impossible; however, the objective clinical outcomes in our analyses are unlikely to be influenced by knowledge of group allocation. We therefore considered the criterion of blinding in terms of outcome assessment (i.e., PSG scoring of AHI). One point was added for a “yes” answer to each of the first five items, and one point was subtracted for a “yes” answer to either of the last two items, for an overall score from 0 to 5.

### Statistical Analysis

All included studies in the primary selection were included in the meta-analysis. Main results were expressed as arithmetic mean and SD. We compared the mean number of steps per day OSA patients to the theoretical threshold of 10,000 steps per day. We also conducted a subgroup analysis by combining the data from studies identified by our systematic review examining the effects of CPAP or exercise/lifestyle interventions on objectively measured physical activity levels by comparing the standardized mean difference of steps per day before and after interventions. Lastly, we calculated the mean exercise training-induced change in AHI, Epworth sleepiness scale, VO2peak, and BMI in the intervention group compared with the control group (26). A DerSimonian and Laird random-effects meta-analysis model was used in each case to combine weighted mean differences (27).

The heterogeneity between studies was measured using the *I*^2^ inconsistency index which provides an estimation of the variability due to the heterogeneity rather than chance. An *I*^2^ index higher than 60% reflects increasing heterogeneity (28).

Finally, the robustness of the results was assessed using sensitivity analysis by leaving out one study at a time, and the absence of selection bias was assessed using funnel plot. The presence or the absence of asymmetry in the funnel plot was assessed using the Egger test. There was no exclusion of studies based on methodological quality assessment results. Meta-analyses were carried out by R package metafor in the RStudio software (RStudio v 1.0.136) (29). A *p*-value threshold of 0.05 was considered for significance.

## Results

### Spontaneous Physical Activity and OSA

The study selection process is presented in Figure 1. The search of Medline and Embase databases provided a total of 2,141 citations. After adjusting for duplicates 1,658 remained. Of these, 1,026 studies were discarded because after reviewing the abstracts it appeared that these papers clearly did not meet the criteria. The full-texts of the remaining 80 citations were examined in more detail. It appeared that 68 studies did not meet the inclusion criteria as described. Fourteen studies met the inclusion criteria and of these, eight studies reported steps per day and were included in the meta-analysis.

#### Main Findings

The characteristics of the eight studies reporting objectively measured steps per day, including 502 participants, are presented in Table 1. The mean number of steps per day across studies was 5,388 (95% CI: 3,831–6,945), which was significantly lower when compared with the recommended threshold of 10,000 steps per day (15) (mean difference is 4,611 steps per day) (Figure 2). The results from the quality assessment using the NOS are presented in Table 2.

**Table 1:** Summary of findings reporting objectively measured steps per day.

Author, year (reference number)	Design	Participants	AHI cutoff, events/h	Sample size	Mean AHI (SD), events/h	Mean age (SD), years	Mean BMI (SD), kg/m^2^	Men, *n*(%)	Measuring device	Wear time	Main findings (steps per day, mean (SD))
Chasens et al. (7)	Regression study	OSA	≥5	37	21.7 (25.4)	49.5 (11.5)	34.0 (7.4)	19 (53)	SenseWear Armband	7 days	6,988 (3,852)
Kline et al. (16)	Baseline data from RCT	OSA	≥15	43	29.3 (4.1)^a^	46.9 (1.2)^a^	34.8 (0.9)^a^	24 (56)^a^	Pedometer NL-1000	7–14 days	5,580 (2,584)
Diamanti et al. (17)	Pre-post CPAP study	OSA	≥15	24	37.5 (22.7)	51.9 (10.6)	34.4 (6.5)	20 (83)	PAL lite	7 days	3,250 (2,327)
Igelström et al. (18)	Regression study	OSA	≥15	63	41.7 (20.9)	55 (12)	35 (5)	58 (80)	SenseWear Armband	4 days	7,734 (3,528)
Verwimp et al. (19)	Regression study	OSA	>20	75	54 (24–108)^b^	51.0 (10.0)	36.0 (7.0)	40 (53)	SenseWear Armband	7 days	6,796 (3,493)
Mendelson et al. (20)	Baseline data from RCT	OSA and high-CDV risk	≥10	107	39 (16.7)	63 (9)	29.9 (4.8)	89 (83)	SenseWear Armband	3 days	7,310 (3,490)
Bamberga et al. (21)	Case–control	OSA	>15	107	32.8 (4.3)	56.1 (3.9)	35.2 (2.0)	N/A	SenseWear Armband	24 h	1,570 (761)
Mendelson et al. (22)	Baseline data from RCT	OSA with CAD	>15	36	31.2 (15.5)	62.6 (9.4)	28.0 (4.3)	30 (88)	Pedometer Omron HJ-320	7 days	4,064 (1,131)

*AHI, apnea–hypopnea index; BMI, body mass index*.

*^a^Data presented by authors as mean ± standard error, which we converted to mean ± SD for the statistical analysis*.

*^b^Data presented by authors as median (min–max)*.

**Table 2:** Quality assessment of *physical activity studies* according to the adapted version of the Newcastle-Ottawa scale.

Author, year (reference number)	Sample selection	Comparability	Evaluation of outcome	Total score
Bamberga et al. (21)	**	**	*	5
Chasens et al. (7)	*	*	**	4
Diamanti et al. (17)	*	*	**	4
Igelström et al. (18)	**	**	*	5
Kline et al. (16)	*	**	**	5
Mendelson et al. (20)	*	**	*	4
Mendelson et al. (22)	*	*	**	4
Verwimp et al. (19)	*	*	**	4

*For the attribution of asterisks, please refer to methodological quality assessment paragraph in the Materials and Methods section*.

**Figure 3:**
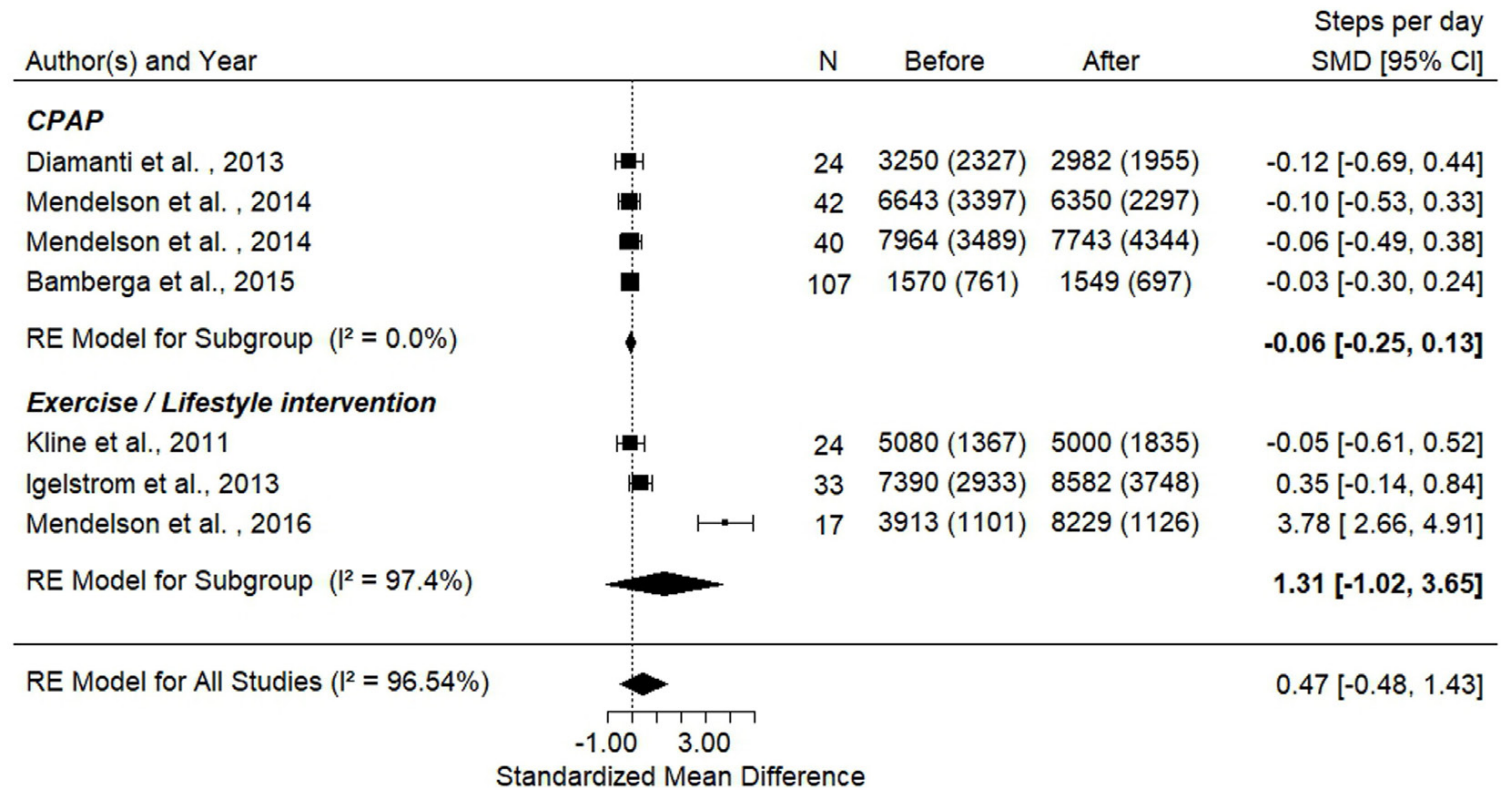
Forest plot presenting steps per day before and after interventions [continuous positive airway pressure (CPAP) and exercise]. Ref (16–18, 20–22).

The results of the subgroup analysis which compared the effects of CPAP or exercise/lifestyle interventions showed no effect of these interventions on objectively measured physical activity levels (Figure 3).

#### Sensitivity Analysis

Sensitivity analyses, done by systematically removing one study at a time, demonstrated that no single study changed the statistical significance of the overall results. The estimation of difference ranks from −4,947 to −4,052 steps per day. The results remain unchanged whether the analysis was performed using a standardized mean or using a logarithm transformation of the number of steps per day. The total heterogeneity of the studies was high (*I*^2^ = 98.65%) and the Egger test showed significant asymmetry in the funnel plot (Figure S1A in Supplementary Material, *p* = 0.047). One study is necessary to correct this asymmetry and is represented by an open circle on Figure S1B in Supplementary Material. However, the results from this sensitivity analysis must be interpreted with caution, since we included a small number of studies.

### Effects of Exercise Training Interventions on OSA

The study selection process is presented in Figure 1B. The search of Medline and Embase databases provided a total of 2,725 citations. After adjusting for duplicates 2,494 remained. Of these, 2,466 studies were discarded because after reviewing the abstracts it appeared that these papers clearly did not meet the criteria. The full-texts of the remaining 28 citations were examined in more detail. It appeared that 22 studies did not meet the inclusion criteria as described. Eight studies met the inclusion criteria, however, authors did not reply to the request to provide data for two articles therefore six studies were included in the meta-analysis.

#### Main Findings

The baseline characteristics including the Jadad score for quality assessment of the trials included in the meta-analysis are presented in Table 3 and the characteristics of exercise interventions are presented in Table S1 in Supplementary Material.

**Table 3:** Baseline characteristics of randomized controlled trials (RCTs) examining the effects of exercise training.

Author, year (reference number)		*n*	Mean AHI (SD), events/h	Mean age (SD), years	Mean BMI (SD), kg/m^2^	Men, *n*(%)	Jadad score
Kline et al. (16)^a^	Exercise group	27	32.2 (5.6)	47.6 (1.3)	35.5 (1.2)	15 (56)	3
	Control group	16	24.4 (5.6)	45.9 (2.2)	33.6 (1.4)	9 (56)	
Sengul et al. (30)	Exercise group	10	15.2 (5.4)	54.4 (6.6)	29.8 (2.7)	10 (100)	0
	Control group	10	17.9 (6.5)	48.0 (7.5)	28.4 (5.4)	10 (100)	
Servantes et al. (31)	Exercise group 1	17	25.5 (24.7)	51.8 (9.8)	26.9 (4.7)	8 (47)	3
	Exercise group 2	17	26.4 (17.6)	50.8 (9.5)	28.0 (4.4)	8 (47)	
	Control group	11	22.8 (17.4)	53.0 (8.2)	27.7 (3.7)	5 (46)	
Ackel-D’Elia et al. (32)	Exercise group	13	40.5 (22.9)	48.4 (9.2)	28.0 (3.1)	13 (100)	1
	Control group	19	42.3 (21.6)	49.5 (7.7)	28.5 (2.2)	19 (100)	
Desplan et al. (33)	Exercise group	11	40.6 (19.4)	Not reported	29.9 (3.4)	Not reported	4
	Control group	11	39.8 (19.2)		31. (2.5)		
Mendelson et al. (22)	Exercise group	17	31.1 (12.9)	63.8 (8.0)	28.6 (4.5)	16 (94)	5
	Control group	17	28.1 (13.5)	59.6 (11.8)	26.2 (3.9)	14 (82)	

*AHI, apnea–hypopnea index; BMI, body mass index*.

*^a^Data presented by author as mean ± standard error, which we converted to mean ± SD for the statistical analysis*.

**Figure 4:**
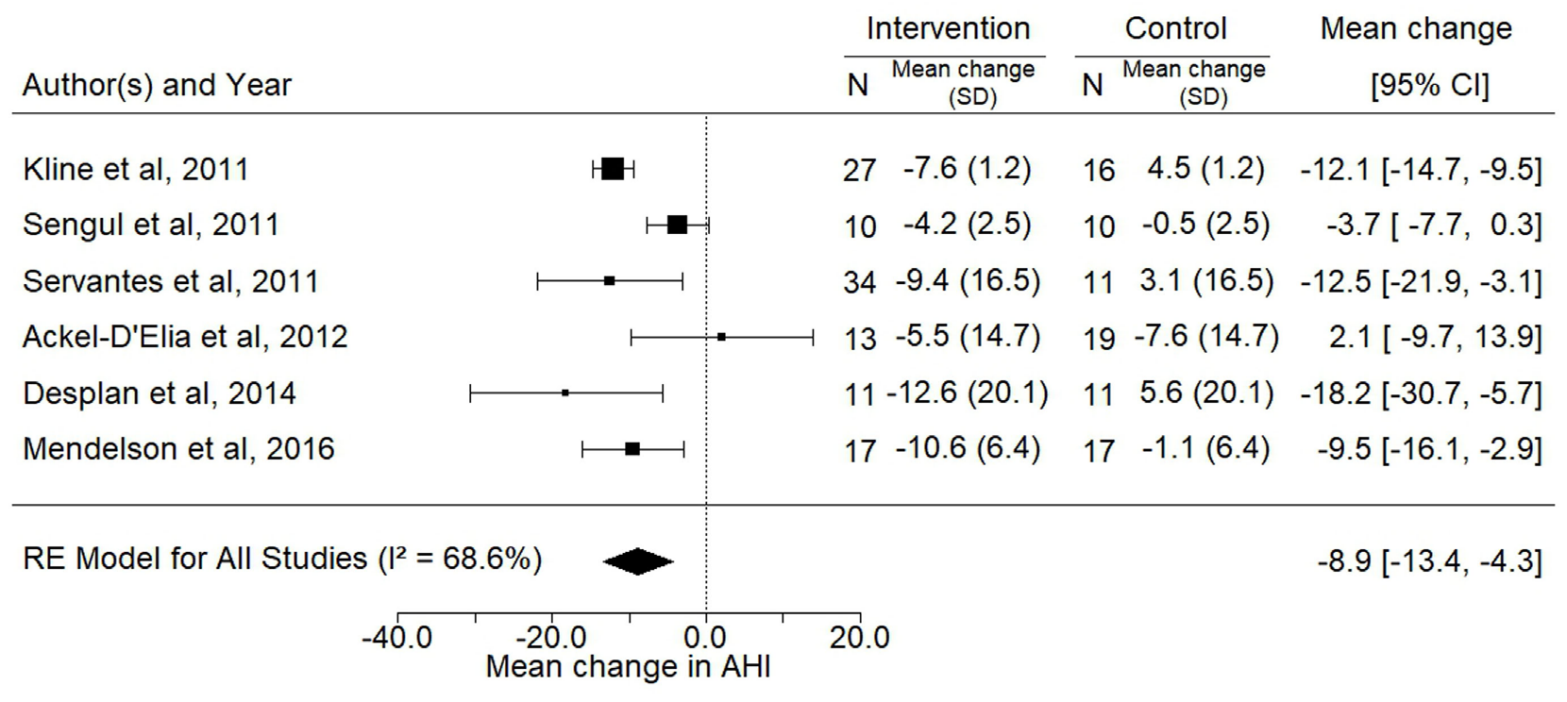
Forest plot for the mean change in apnea–hypopnea index (AHI) (events/h) following exercise training. The diamond reflects the 95% confidence interval of the pooled estimate of mean difference. Ref (16, 22, 30–33).

There was a significant decrease in AHI in the intervention arm compared with the control arm. A mean reduction of 8.9 (−13.4; −4.3) events/h (*p* < 0.01), which reflected a 28% reduction in AHI from baseline was detected in the exercise intervention groups (Figure 4).

**Figure 5:**
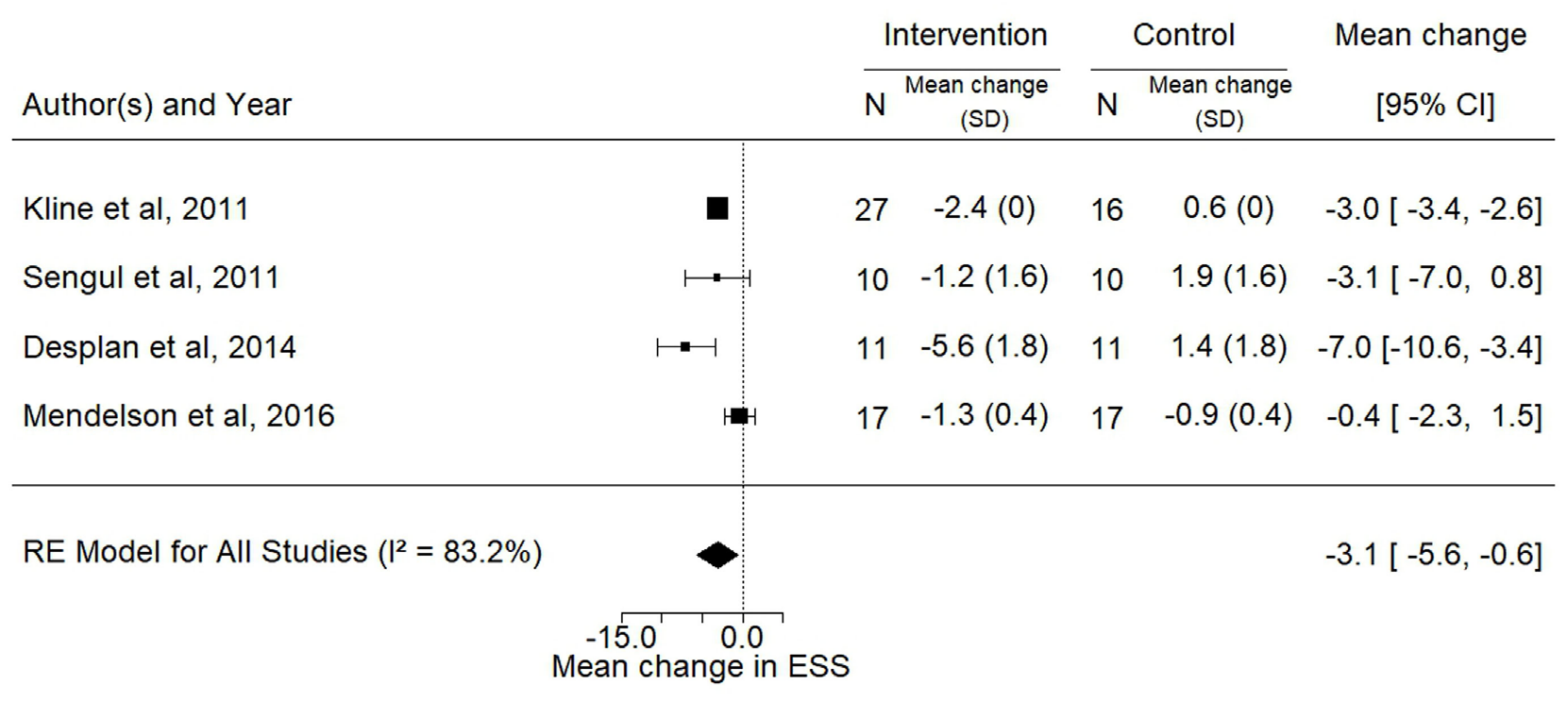
Forest plot for the mean change in Epworth sleepiness scale following exercise training. The diamond reflects the 95% confidence interval of the pooled estimate of mean difference. Ref (16, 22, 30, 33).

**Figure 6:**
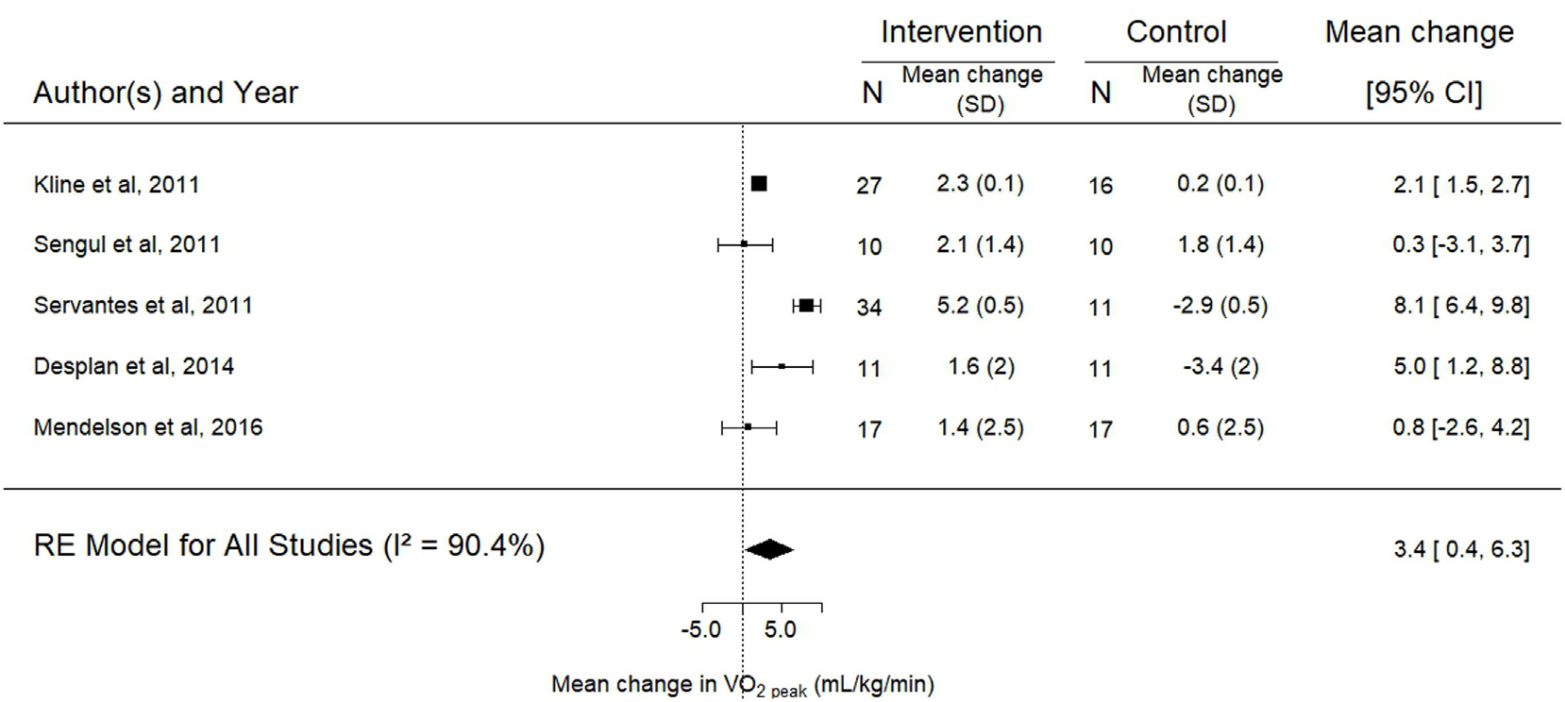
Forest plot for the mean change in peak oxygen consumption (VO2peak) following exercise training. The diamond reflects the 95% confidence interval of the pooled estimate of mean difference. VO2peak measured in milliliter per kilogram per minute. Ref (16, 22, 30, 31, 33).

**Figure 7:**
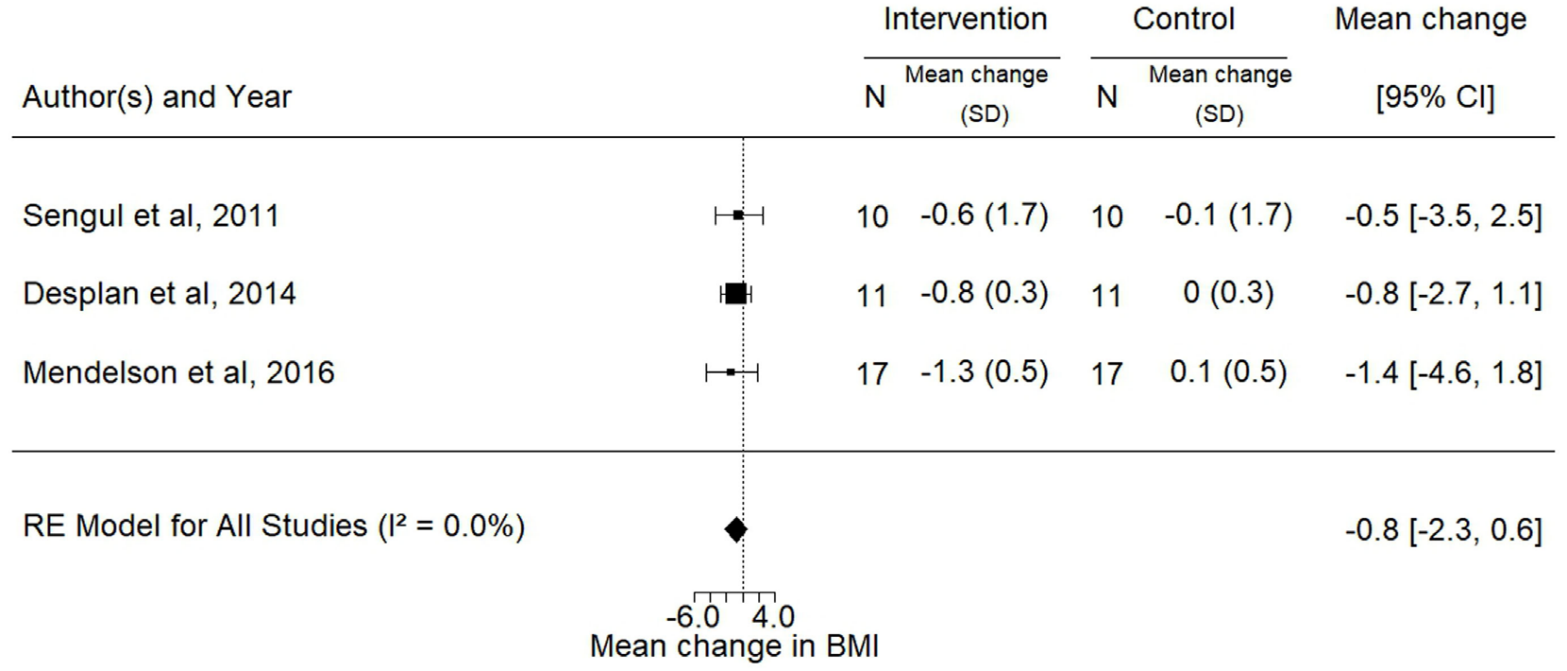
Forest plot for the mean change in body mass index (BMI) (kilogram per square meter) following exercise training. The diamond reflects the 95% confidence interval of the pooled estimate of mean difference. Ref (22, 30, 33).

Data for the effects of exercise training on BMI, ESS, and VO2peak were available for three, four, and five trials, respectively. The analyses showed significant improvements in the mean ESS (−3.1; 95% CI −5.6 to −0.6; *p* = 0.02) and VO2peak (3.4 mL/kg/min; 95% CI 0.4–6.3, *p* = 0.03) after intervention while no change was observed in BMI (*p* = 0.24) (Figures 5–7).

#### Sensitivity Analysis

A sensitivity analysis done by using a leave and out method demonstrated no major change in the overall results.

## Discussion

### Spontaneous Physical Activity and OSA

Based on a systematic review and meta-analysis of the reported findings, OSA patients present low levels of objectively measured physical activity when compared with public health physical activity guidelines (15, 34).

The mean BMI of the OSA patients included in the meta-analysis was over 30 kg/m^2^ for six out of the eight studies included, indicating that a majority of the patients were obese. This could have contributed to the low level of physical activity observed in these patients. A recent paper examining physical activity levels in patients with varying levels of obesity found that physical activity levels tended to be lower with increasing obesity (35). Patients with class III obesity performed on average 6,617 ± 2,673 steps per day, which was significantly lower than physical activity levels achieved in lean participants (9,951 ± 3,487 steps per day). Even if undiagnosed OSA may be present in some of the overweight/obese patients investigated in this previous study due to the relationship between weight status and risk of OSA (36), the average steps per day reported was over 1,000 steps higher than the mean values obtained in the present meta-analysis. This suggests that OSA *per se* is associated with a significant reduction in physical activity levels.

Furthermore, mean physical activity levels have been reported in other chronic health conditions such as chronic obstructive pulmonary disease (COPD) and coronary artery disease. For example, one study in 343 patients with COPD showed that the mean number of steps per day was approximately 5,000 (37). In 64 patients with coronary artery disease, mean number of steps per day was 5,191 ± 3,198 (38). These results are similar to what we observed in patients with OSA, suggesting that OSA reduces physical activity levels to a similar extent than other chronic health conditions.

The results of our meta-analysis provide interesting observational data but do not provide insight as to what are the limiting factors to performing physical activity in OSA patients. The studies identified by our systematic review provide conflicting data with respect to the relationship between OSA severity and physical activity levels. In patients with OSA and high-cardiovascular risk, physical activity levels were shown to be inversely related to BMI but not to the AHI (39), suggesting that weight status has an important effect on physical activity levels. Conversely, Chasens et al. (7) found that increased OSA severity was associated with decreased objectively measured physical activity after controlling for age, sex, and ESS. Verwimp et al. (19) found that only AHI in rapid-eye-movement sleep was independently associated with steps per day after controlling for age and BMI. Furthermore, one study included patients with coronary artery disease and OSA (22) and in another study over 60% of the patients were in secondary prevention (20), therefore it is possible that physical activity was limited in this population due to the cardiovascular co-morbidity.

The impact of OSA on exercise tolerance remains unclear. Previous studies have yielded conflicting results, with some demonstrating a reduction in cardiorespiratory fitness (i.e., VO2peak) (40–43) and others suggesting that cardiorespiratory fitness is not impaired (44–46). These studies have reported different physiological adaptations during exercise, such as exaggerated blood pressure, delayed heart rate recovery, and chronotropic incompetence. Therefore, it is also possible that symptoms of exercise intolerance limit spontaneous physical activity levels of patients with OSA.

**Figure 8:**
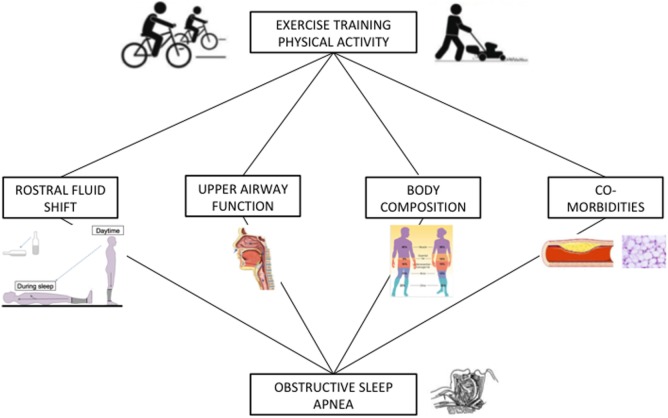
Hypothetical relationship between exercise training/physical activity and obstructive sleep apnea (OSA). The rostral fluid shift contributes to the pathogenesis of OSA and its attenuation *via* physical activity (47) and exercise training has been shown to alleviate OSA. The strength and fatigability of the upper airway dilators have been shown to be altered in patients with OSA. Specific exercise training modalities may improve upper airway function in OSA patients and thus contribute to decrease OSA severity. An elevated body mass index (BMI) is a key risk factor for the development of OSA while sleep disturbances can influence body composition. Exercise training has been shown to favorably modify body composition (increase lean mass, decrease fat mass) and can reduce BMI, therefore potentially alleviating the severity OSA. OSA is often accompanied by cardiovascular and metabolic co-morbidities, which can impair exercise tolerance. Exercise training has been shown to be beneficial for the improvement of a number of these co-morbidities (hypertension, dyslipidemia, type 2 diabetes, etc.).

Taken together, these results suggest that a number of factors can influence physical activity levels in OSA patients, such as weight status, severity of sleep apnea, the presence of co-morbidities, and exercise tolerance (Figure 8). To date, no study has compared objectively measured physical activity levels in OSA patients with age, BMI, and gender-matched controls. Therefore future controlled studies should bring further insight to objectively measured physical activity levels and its determinants in patients with OSA.

#### Effects of CPAP or Exercise Interventions on Physical Activity

In a subgroup analysis, we found that CPAP did not impact physical activity levels. One would hypothesize that treatment of OSA with CPAP, which reduces daytime sleepiness and improves quality of life, would encourage patients to be more active. However, in all the studies published to date this was not observed. For example, in a randomized trial, West et al.’s showed that 3 months of CPAP had no effect on objective physical activity levels, measured in arbitrary units (48). In the studies included in this subgroup analysis, OSA patients were either overweight (*n* = 3) or obese (*n* = 4). Therefore, physical activity may be limited by weight status despite improvements in symptoms of fatigue and sleepiness. Interestingly, a recent meta-analysis demonstrated that treatment of OSA with CPAP promoted a significant increase in BMI and body weight (49). In line with these results, chronic intermittent hypoxia, a key component of OSA, has been shown to promote weight loss in mice (50) and sympathetic overactivity, another hallmark of OSA, promotes increases in energy expenditure. Hence, the reversal of OSA using CPAP may potentially result in a small but significant increase of BMI and body weight (49), which may contribute to limiting the increase in physical activity levels. The lack of effect of CPAP on physical activity levels may also suggest that it is not only the fatigue and sleepiness often reported by OSA patients that impact their activity levels but other factors that CPAP fails to improve such as established poor lifestyle habits and co-morbidities (Figure 8).

The disappointing results of these trials suggest that improving sleep and sleep apnea is insufficient to change physical activity. Furthermore, to explore the role of weight status on physical activity levels in OSA patients, future investigations evaluating the effects of OSA treatment should aim to differentiate the effects in lean versus obese patients.

Three randomized trials examined the effects of an exercise (16, 22) or a lifestyle intervention (51) on physical activity levels. Only one trial found increased physical activity levels after intervention in patients with OSA and coronary artery disease (22). These patients completed a walking-based short-term exercise training trial (4 weeks) prior to entering cardiac rehabilitation. After completing the trial, it is likely that the maintenance of their increased levels of physical activity was facilitated by the participation in a structured exercise program. The results from the two other trials in which the interventions did not result in increased physical activity levels are more frequently observed and are consistent with the observation that following rehabilitation, objectively measured physical activity levels do not change in different patient populations (52, 53). This result is noteworthy because it highlights the importance of long-term maintenance of increased physical activity levels. Indeed, the long-term success of exercise training or lifestyle interventions rests in part on the patient’s ability to maintain the health behaviors acquired following the end of formal treatment. In both pulmonary and cardiac rehabilitation, it has been shown that longer rehabilitation programs (i.e., >12 weeks) may have greater impacts on physical activity levels (52, 53). Furthermore, physical activity coaching interventions which can include accurate assessment and feedback of physical activity levels, individualized physical activity goals and/or tailored motivational messages have shown promise in COPD for maintaining physical activity levels (52).

Future studies identifying and characterizing subgroups of OSA patients with different patterns of change in physical activity levels after interventions may help determine which patients could potentially benefit the most from interventions targeting physical activity levels. Furthermore, interventions should be designed with long-term maintenance of physical activity levels in mind. As mentioned previously, longer programs have been shown to be more effective in different patient populations (52, 53) and specific maintenance programs which include objective monitoring of physical activity levels are likely necessary in OSA patients to facilitate a change in behavior. These types of interventions should be explored in patients with OSA.

#### Methodological Aspects

Methodological aspects must be taken into account when interpreting these results. We chose to focus on objective measurements of physical activity because they are preferred to subjective, questionnaire-based assessments. Physical activity monitors are being frequently used to objectively measure daily levels of physical activity in a wide range of patient populations. These devices can be uniaxial, biaxial, or triaxial and use piezoelectric accelerometers. The signal obtained can then be transformed into an estimate of energy expenditure, summarized as activity counts, or expressed as steps per day (54). The availability of physical activity monitors has made the objective evaluation of physical activity in OSA patients possible in various contexts such as in response to CPAP (17, 20, 48) and during rehabilitation programs (22, 33).

An important source of heterogeneity across studies is the different procedures of data processing. In fact, each manufacturer of different accelerometer devices has its own approach to filter, amplify, or convert the acceleration signals into an output value, either activity counts, steps per day or another variable. Therefore, these data are not always comparable between devices (55).

The site of measurement (i.e., hip, upper arm, or wrist) varies between devices and can also influence the quality of the measure. Current recommendations suggest wearing the device at the hip (i.e., as close as possible to the center of mass) (56).

Furthermore, ideally, physical activity monitoring needs to be done over several days in order to get a good representation concerning spontaneous physical activity levels. While it has been shown that at least 3 days is sufficient in the elderly (57), it seems that in younger patients a full week of monitoring is advisable due to variations in weekly patterns. In the present meta-analysis, physical activity monitoring was carried out over 7 days in five out of eight studies. We took this factor into account when assessing the quality of the studies.

Lastly, to improve the accuracy and precision of physical activity measures, it has been suggested to combine heart rate monitoring with accelerometer-based data (58).

### Effects of Exercise Training Interventions on OSA

The present meta-analysis represents the most recent literature on studies examining the effects of exercise training on OSA. We focused on RCTs examining the effects of exercise as the main intervention. The results obtained suggest that exercise training can reduce the severity of OSA by 28% and improve symptoms of sleepiness and VO2peak, in the absence of any change of BMI, which supports the results of two previous meta-analysis’ (59, 60).

The reduction of OSA severity was observed in the absence of a significant decrease in BMI which suggests a potential role of exercise training in the treatment of sleep apnea.

The precise mechanisms underlying the improvement in AHI following exercise training have not been entirely elucidated (Figure 8). One potential mechanism was explored *via* an RCT included in our meta-analysis, which concomitantly measured the rostral fluid shift in the participants. Mendelson et al. (22) found a reduction in the overnight change in leg fluid volume as well as an increase in the upper airway cross-sectional area. Other potential mechanisms which have been suggested to explain the observed decrease in OSA severity following exercise training include increased strength and fatigue resistance of the upper airway dilators, decreased nasal resistance, and increased respiratory stability through deeper sleep (16). Finally, even though it is not consistently reported, body composition modification, and changes in fat mass distribution with exercise training may also contribute to sleep apnea improvement (33).

The AHI reduction observed following exercise training in the present study is lower than results obtained in previous meta-analyses examining the effects of weight loss (61), bariatric surgery (62), oropharyngeal exercises (63), oral mandibular appliances and CPAP (64) on OSA severity. Future investigations should aim to define specific rehabilitation strategies for the different OSA phenotypes (65).

Besides improvement in the AHI, exercise training also improved VO2peak. This improvement is noteworthy as it is associated with a significant survival benefit (66). A meta-analysis summarizing the relationship between mortality and cardiorespiratory fitness showed that a 1-metabolic equivalent higher level of maximal aerobic capacity was associated with 13 and 15% decrements in risk of all-cause mortality and coronary heart disease/cardiovascular disease, respectively [1 MET corresponds to 3.5 mL//kg min of oxygen consumption, VO2, according to the Metabolic Calculation Handbook by the American College of Sports Medicine (67)]. In the present meta-analysis, we observed a 3.4 mL/kg/min increase in VO2peak after exercise training in OSA patients. This result is clinically significant since CPAP does not prevent cardiovascular events in patients with moderate-to-severe OSA and established cardiovascular disease (68) and emphasizes the potential role of exercise as an adjunct treatment to CPAP.

In the studies included in the present meta-analysis, authors did not investigate which exercise training modality (walking versus cycling), exercise intensity (high versus moderate versus low) and duration is the most effective for patients with OSA. Future investigations should further explore this question.

#### Effects of Exercise Training on Co-Morbidities/Cardiovascular Risk Factors in OSA Patients

Interestingly, only one study identified by our systematic review examined the effects of exercise training on outcomes other than those directly related to sleep apnea. This is surprising given that CPAP, the first line treatment of OSA has a limited impact on cardiometabolic risk factors (69) while as exercise training has a well-documented effect on a number of cardiometabolic risk factors in non-OSA patients (70).

Desplan et al. (33) examined the effects of a 4-week comprehensive rehabilitation program on metabolic syndrome components. For instance, they observed a 4 mmHg reduction in diastolic blood pressure, a 15 mg/dL decrease in fasting glucose, and a decrease in the prevalence of metabolic syndrome (from *n* = 10 to *n* = 6) in patients with OSA.

Regular exercise is considered a cornerstone in the prevention and management of hypertension and cardiovascular disease (71). Results from a meta-analysis examining the effects of endurance exercise training on blood pressure and cardiovascular risk factors in patients with and without hypertension showed a 6.9 mmHg reduction in systolic blood pressure and 4.9 reduction in diastolic blood pressure in hypertensive patients (72). A reduction of this magnitude in blood pressure is clinically relevant since a 5 mmHg reduction in population average resting systolic blood pressure can reduce all-cause mortality by approximately 7% (73). Exercise has also been shown to improve glycemic control (74) and insulin sensitivity (75) in patients with type 2 diabetes. This is important because it has been shown that OSA is associated with insulin resistance, glucose intolerance, and type 2 diabetes, independent of obesity (76). Furthermore, a meta-analysis on the effects of aerobic exercise training has shown improved HDL-C and minimal changes in LDL-C level. However, a substantial decrease in TC and LDL-C and significant improvement in HDL-C levels are observed when a reduction in body weight following exercise training is achieved (77).

Future well-designed studies comparing a combination of exercise training and sleep apnea treatment (mandibular advancement device or CPAP) which focus on long-term cardiovascular outcomes in OSA patients are needed.

### Strategies to Enhance Physical Activity in OSAS

Low-cost wearable devices and mobile devices that assist individuals in monitoring their physical activity levels and becoming more active have become more widespread (78). These devices have been used in clinical studies of patients receiving rehabilitation or treatment for chronic diseases such as osteoarthritis, chronic heart failure, diabetes, peripheral neuropathy, or COPD (79–82).

Physical activity is a multidimensional and complex health behavior, therefore interventions aiming to improve physical activity levels need to reflect this. Recent data on the use of mobile devices for increasing physical activity has shown that these platforms are effective means for influencing physical activity behavior (83). New generations of physical activity monitors have the potential to provide direct feedback to the patient, which has yielded encouraging results when used in combination with goal setting in various patient populations, such as COPD (84) and obesity (85).

A large multicenter randomized trial in patients with COPD evaluating a semiautomated tele-coaching intervention using a pedometer and an application installed on a smartphone showed significant increases in physical activity levels (37).

The use of mobile technology and physical activity tracking is still a relatively recent field, however, the available data to date can be the basis for developing targeted and efficiently interventions using this technology to increase physical activity levels.

## Limitations

The current meta-analysis presents the strength of combining data across a number of studies in order to estimate objectively measured physical activity levels and the effects of structured exercise training intervention in OSA patients with more precision than is possible in a single study. We chose to focus on steps per day because this measure was the most frequently reported outcome across all the studies we identified. However, it is important to note that step count is just a component of the spectrum of physical activity and does not reflect the complete activity being performed by a patient.

Our meta-analysis had a limited number of studies and this limits the generalization of our results. There was also high heterogeneity in the included studies and due to their small number; we were unable to further investigate this heterogeneity.

The interpretation of the current meta-analysis may also be limited by publication bias (i.e., probability of unreported studies).

## Conclusion

Based on a systematic review and meta-analysis of the reported findings, OSA patients present low levels of objectively measured physical activity. These low levels of physical activity may contribute to obesity and cardiovascular risk within OSA patients. Therefore, improving physical activity levels should be considered as a key component in the management of patients with OSA. CPAP improves symptoms and quality of life in symptomatic OSA patients but has a limited impact when prescribed for increasing physical activity levels. Increasing physical activity (with structured exercise training programs for instance) improves OSA indices and may also reduce important cardiometabolic risk factors associated with OSA. Hence, there is a strong need for combined treatment strategies for OSA patients with the aim of optimizing risk factor control and reducing long-term morbi-mortality. Nevertheless, more research is needed to further clarify the potential therapeutic role of exercise in the treatment of OSA. For example, future studies should identify the subset of patients most likely to respond to exercise training and to adhere to this type of treatment in the long-term as well as determine the most efficient exercise training modalities and the optimal strategies to enhance spontaneous physical activity.

## Author Contributions

MoniqueM, SB, MathieuM, PF, J-CB, IV, SD, SV, RT, and J-LP study conception and design. MoniqueM, SB, MathieuM, and SV acquisition of data. MoniqueM, SB, MathieuM, SV, PF, J-CB, IV, SD, RT, and J-LP analysis and interpretation of data. MoniqueM, SB, MathieuM, SV, and J-LP drafting of manuscript. MoniqueM, SB, MathieuM, PF, J-CB, IV, SD, SV, RT, and J-LP critical revision.

## Conflict of Interest Statement

The authors declare that the research was conducted in the absence of any commercial or financial relationships that could be construed as a potential conflict of interest.

## Supplementary Material

Figure S1 Funnel spot for studies reporting steps per day.

## Abbreviations

AHI, apnea–hypopnea index; BMI, body mass index; CPAP, continuous positive airway pressure; ESS, Epworth sleepiness scale; OSA, obstructive sleep apnea; RCT, randomized controlled trial; VO2peak, peak oxygen consumption.
