# Supplementary figures and images for: Obstructive Sleep Apnea Syndrome, Objectively Measured Physical Activity and Exercise Training Interventions: A Systematic Review and Meta-Analysis
Source: Front Neurol. 2018 Feb 22;9:73. doi: 10.3389/fneur.2018.00073 (PMC5827163; doi:10.3389/fneur.2018.00073)

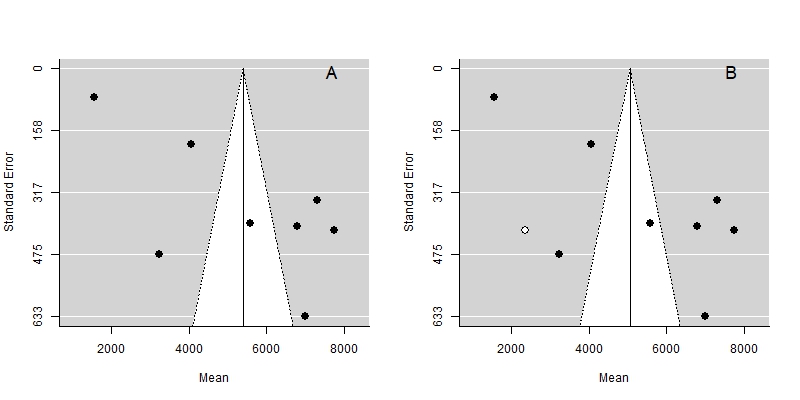

Supplement: Figure S1 — Funnel spot for studies reporting steps per day. [file image_1.jpeg]
